# Neurofilament Light Chain as a Biomarker for Monitoring the Efficacy of Transcranial Magnetic Stimulation on Alcohol Use Disorder

**DOI:** 10.3389/fnbeh.2022.831901

**Published:** 2022-02-07

**Authors:** Tian Zhang, Bin Song, Yanfei Li, Ranran Duan, Zhe Gong, Lijun Jing, Kaixin Wang, Bingquan Ma, Yanjie Jia

**Affiliations:** ^1^Department of Rehabilitation, First Affiliated Hospital of Zhengzhou University, Zhengzhou, China; ^2^Department of Neurology, First Affiliated Hospital of Zhengzhou University, Zhengzhou, China

**Keywords:** NfL, alcohol dependence, TMS, biomarker, neuropsychological assessment, alcohol use disorder, neurofilament light chain, transcranial magnetic stimulation

## Abstract

**Objective:**

This study assessed the effects of repetitive transcranial magnetic stimulation (rTMS) of the left dorsolateral prefrontal cortex (DLPFC) on serum neurofilament light chain (NfL) levels, alcohol consumption, craving, and psychological impairment in participants with alcohol use disorder (AUD).

**Methods:**

Participants with AUD were randomly assigned to receive one of two treatments (active or sham rTMS). All participants received 10 daily active or sham rTMS sessions over the left DLPFC for 2 weeks, with follow-up visits at baseline and immediately after the completion of the treatments. Serum samples were obtained before and after the intervention. Days of heavy drinking, visual analog scale (VAS) scores, and mental health component scores (MCSs) of the Medical Outcomes Study 36-Item Short Form Health Survey were used to assess the effects of rTMS.

**Results:**

Active rTMS had a significant effect on reducing days of heavy drinking, alcohol craving, and serum NfL levels, and improved social functioning and mental health. The improvement with active rTMS was significantly greater than that with sham rTMS. Correlation analysis revealed that the reduction in the baseline drinking level was positively correlated with declines in the VAS and NfL levels but not with psychological scores.

**Conclusion:**

Repetitive transcranial magnetic stimulation of the left DLPFC was associated with reducing alcohol consumption and craving in patients with AUD and positively impacted neuropsychological and social function. Serum NfL levels may be useful as an early serological indicator of alcohol-induced brain injury.

## Introduction

Alcohol is the most widely used psychoactive substance. However, alcohol use disorder (AUD) can be extremely harmful to humans. In the Diagnostic and Statistical Manual for Mental Disorders, 5th edition (DSM-V; American Psychiatric Association, 2013), AUD is defined as a pattern of alcohol consumption that results in problems associated with two or more of eleven potential symptoms of AUD. AUD is a substantial public health issue (Grant et al., 2015). Based on data from the World Health Organization, AUD is one of the top five leading causes of death and disability worldwide (Peacock et al., 2018). It is a chronic relapsing brain disease that is highly comorbid with several mental health conditions, including depression, anxiety, and personality disorders (World Health Organization [WHO], 2019a). Excessive drinking can impair social cognition in drinking individuals, which can cause social function impairment (World Health Organization [WHO], 2019b). One or more of these problems may cause either a failure in alcohol withdrawal or the development and maintenance of alcohol addiction (Foster et al., 1999). Abstainers are likely to resume drinking to relieve anxiety, depression, and other negative emotions. Investigators have reported negative emotions (e.g., depression and anxiety) or social stress account for about 70% of relapse cases after abstinence (Zywiak et al., 2003). Improving the mental health of individuals with AUD may help maintain abstinence and prevent relapse. Therefore, one or more effective, acceptable, and safe methods are urgently needed to solve these problems in the AUD population.

Only three drugs have been approved by the United States Food and Drug Administration (FDA) to treat AUD; other drugs were approved in Europe (nalmefene in Europe by EMA, sodium oxybate by AIFS in Italy and by AGES in Austria and baclofen by ANSM in France). However, the adverse effects of pharmacological treatments cannot be ignored (Akbar et al., 2018). Neuromodulation techniques are expected to bring new hope for developing novel treatments for AUD (Witkiewitz et al., 2019). In recent years, great progress has been made to understand the central mechanisms underlying alcohol addiction (Costardi et al., 2015). However, translating knowledge into evidence-based clinical practice is urgently needed (Ekhtiari and Paulus, 2016). In recent reviews, repetitive transcranial magnetic stimulation (rTMS) has been considered a promising treatment strategy for AUDs (Ekhtiari et al., 2019; Philip et al., 2020; Turco et al., 2020; Antonelli et al., 2021). However, a consensus has not been formulated because of methodological differences among the studies (Costardi et al., 2015). Substance-dependent individuals are prone to craving and drug-seeking behavior caused by substance-related cues. This behavior may occur because of impaired executive control and limbic system function caused by abnormal activity in multiple parts of the prefrontal lobe-striatum circuit (Hanlon et al., 2015).

It is widely accepted that the dorsolateral prefrontal cortex (DLPFC) is a major target brain region in AUD; the choice between the left and the right DLPFC remains controversial and requires further in-depth investigations (Zhang et al., 2019). However, rTMS has been approved by the FDA as an alternative treatment for severe depression (Perera et al., 2016). Strong evidence from research supports the effectiveness of high-frequency TMS treatment of the left DLPFC in depression (Lisanby et al., 2009; Schutter, 2009; George and Post, 2011; Solvason et al., 2014). In addition, some scholars have found a mechanistic link between excessive right DLPFC activity and the expression of physiological anxiety (Balderston et al., 2020). Therefore, whether the left DLPFC can be the main target of rTMS therapy in AUD patients with anxiety, depression, and other psychological problems is worth further discussion.

Heavy chronic or binge alcohol exposure can cause severe adverse effects on brain structure and function. It has been reported that white matter volume is significantly reduced in the brains of long-standing heavy alcoholics (Kril et al., 1997). Furthermore, excessive alcohol consumption can also cause neuroinflammation, toxicity, and synaptic dysplasia (de la Monte and Kril, 2014). Neurofilaments [which consist of neurofilament heavy chain (NfH), neurofilament medium chain (NfM), and neurofilament light chain (NfL)], are intermediate filaments of the cytoplasmic scaffold that make up the axon cytoskeleton (Fuchs and Cleveland, 1998). Recent studies suggest that axonal injury or degeneration in central nervous system diseases increases NfL concentrations in the cerebrospinal fluid and blood (Li et al., 2019; Lin et al., 2019; Olsson et al., 2019). In several inflammatory, neurodegenerative, traumatic, and cerebrovascular conditions, NfL levels in the cerebrospinal fluid and blood increase in proportion to the extent of axonal injury (Khalil et al., 2018). Compared to standard magnetic resonance imaging (MRI) and the relapse rate, NfL measurement may provide a more sensitive and accurate picture of the extent of ongoing axonal damage in normal-appearing white matter (Tallantyre et al., 2010). Investigators have observed that the repeated detection of NfL in peripheral blood to detect axonal injury may be a new method to monitor disease activity and treatment effects in multiple sclerosis (Novakova et al., 2017). In a previous study by our research team, we found that individuals with AUD had higher NfL levels than healthy controls (Li et al., 2021); however, relevant reports are lacking regarding whether the detection of NfL can be used as a biomarker for monitoring the response to therapy in AUD.

This study evaluated the efficacy of applying rTMS on the left DLPFC to treat participants with AUD. We also measured the expression of serum NfL in individuals with AUD and evaluated the alteration in alcohol consumption, craving, and psychological impairment before and after treatment.

## Materials and Methods

### Study Design

This study was a double-blind, randomized, controlled trial conducted at the First Affiliated Hospital of Zhengzhou University (Zhengzhou, Henan Province, China). Participants received 10 daily rTMS sessions over the left DLPFC for 2 weeks with a follow-up visit immediately after the completion of the treatments, including assessment of self-reported alcohol consumption and craving, psychological health, and NfL levels. The First Affiliated Hospital of Zhengzhou University Institutional Review Committee approved all research procedures. All participants or their guardians provided written informed consent.

### Participants

We enrolled 48 individuals with a history of heavy drinking in this clinical trial between March 2019 and October 2021. Recruitment relied on public media advertisements, online postings, and word-of-mouth with the inclusion and exclusion criteria (Table 1).

**TABLE 1:** Study inclusion and exclusion criteria.

Inclusion criteria	Exclusion criteria
Between the ages of 18 and 65 years old	Acute alcohol withdrawal (CIWA-Ar > 9)
Meets the diagnostic criteria for AD according to the DSM-V	Severe neurological or psychiatric disorders due to a medical condition other than alcohol dependence, such as stroke, intracranial infection, brain tumor, schizophrenia, severe depressive disorder, bipolar disorder, etc.
No history of major neurological or psychiatric illness	Experienced traumatic brain injury or other damage to brain tissue
Volunteered for the study and willing to cooperate with follow-up observation	Using any other psychotropic substance, or dependent on any other drug or substance
	Contraindication to TMS pacemaker (e.g., acute infectious disease, implanted pacemaker stimulator, history of epilepsy, metallic craniofacial implants, pregnancy, etc.)

*AD, alcohol dependence; DSM-V, Diagnostic and Statistical Manual of Mental Disorders 5th edition; CIWA-Ar, Clinical Institute of Withdrawal Assessment of Alcohol Scale, Revised; TMS, transcranial magnetic stimulation.*

### Screening, Randomization, and General Procedures

Participants who met the selection criteria were first interviewed and were required to sign an informed consent form. The assessments included age, sex, medical history, physical examination, TMS adult safety screening questionnaire, and contraindications to TMS and MRI. In addition, we gave each participant a free high-resolution MRI scan. A randomization list was generated from randomly arranged blocks, and participants were assigned to one of two treatments (active rTMS or sham rTMS) in the order in which they entered the study. The participants were assigned to the two groups in a 2:1 (active:sham) ratio.

### Transcranial Magnetic Stimulation Procedures

As this study evaluated the feasibility, safety, and tolerability of TMS in the treatment of AUD, all participants were asked to abstain from alcohol and record any new or increased discomfort throughout the treatment.

#### Determining the Resting Motor Threshold

The resting motor threshold (rMT) was determined for all participants before the 1st and 6th TMS treatments. rMT refers to the minimum stimulus intensity required to generate a motor-evoked potential exceeding 50 μV in at least five of 10 consecutive trials at rest (Rossini et al., 2015). The coils were placed in the motor cortex and fine-tuned until each pulse induced an isolated abduction of the right thumb. The intensity of the magnetic pulse was adjusted until the minimum intensity that generated thumb movement occurred 50% of the time (Ziemann and Hallett, 2000).

#### Targeting of the Left Dorsolateral Prefrontal Cortex

Repetitive transcranial magnetic stimulation was delivered using a CCY-2 Magstim Rapid device (Yiruide Medical Equipment Company Ltd., Wuhan, Hubei, China) with an air-cooled figure-8 coil with the handle pointing backward. The motor cortex target (M1) is the position of the coil that we used for the rMT assessment. To determine the approximate position of the left DLPFC, we moved the TMS coil 6 cm in front of the M1 region along a line parallel to the sagittal line (Herbsman et al., 2009; George and Post, 2011). After the first visit, we used the MagVenture TMS reduction cap (MagVenture, Inc., Alpharetta, GA, United States) to copy the treatment site when the participants visited.

#### Active Repetitive Transcranial Magnetic Stimulation Procedure

Participants received 10 sessions of high-frequency rTMS treatment on the left DLPFC five times weekly. Treatment was standardized at 80–110% of the rMT, at 20 pulses per second (20 Hz) for 5 s, with an inter-train interval of 15 s. Treatment sessions lasted for 10 min with 2,000 pulses per session. The treatment parameters for this study were based on previously published data on craving reduction by using rTMS (Herremans et al., 2013, 2015; Ceccanti et al., 2015; Girardi et al., 2015).

#### Sham Repetitive Transcranial Magnetic Stimulation Procedure

We used a device consisting of a coil that looked and sounded similar to the rTMS coil and a transcutaneous electrical nerve stimulator (TENS) device. The TENS device produced a small amount of electrical stimulation on the scalp below the hairline to simulate a response to effective rTMS (Borckardt et al., 2008). Active rTMS was provided free of charge to all sham rTMS participants at the end of the follow-up period.

#### Assessment Procedures

Participants were assessed at baseline (T0) and immediately after the last rTMS session (T1). The assessment included alcohol consumption, craving, and psychological health. The participants and evaluators were blinded to the rTMS stimulation condition.

### Assessment Instruments

#### Alcohol Consumption and Craving

Participants were asked to record their alcohol intake by using self-reports. The records included the type and number of alcoholic beverages consumed each day in the previous week, the number of days consumed, the amount of alcohol contained in each glass, etc., to calculate the number of standard alcoholic beverages consumed on a given day. Finally, heavy drinking days per week were calculated (heavy drinking was defined as ≥4 drinks per occasion for women and ≥5 drinks per occasion for men). When using the visual analog scale (VAS), participants were asked to mark a position on a continuous line between two endpoints that represented their degree of agreement with the statement. In this study, drinking cravings were represented using two endpoints (no drinking cravings:0, and very strong drinking cravings:10) at the time of the evaluation.

#### Psychological Health

The Medical Outcomes Study 36-Item Short Form Health Survey (SF-36) (Ware and Sherbourne, 1992) was developed by the Boston Institute of Health (Boston, MA, United States) and was included in the International Quality Of Life Assessment Project in 1991. The Mental Health Component Score (MCS; Ware et al., 1994, 1995) from the Chinese version of the SF-36, translated at the Department of Social Medicine in Zhejiang University, was used to assess the participants’ social and psychological conditions. The MCS consists of four subscales: vitality, social functioning, role-emotional, and mental health; scored from 0 to 100, with higher scores indicating better overall mental health functioning. It has been verified by the data source and is considered to have excellent reliability (Jenkinson et al., 1997).

### Neurofilament Detection

Fasting blood samples were obtained on the morning of the first TMS treatment day pre-intervention. For the post-intervention assessment, serum was collected 10 weeks after the last intervention. Peripheral blood samples were obtained by venipuncture during the clinical assessments. Serum was obtained by the centrifugation of blood samples at 200 × *g* for 20 min. The supernatants were collected into 1.5-mL polypropylene tubes, and the samples were transported on dry ice. The concentration of NFL was detected by Guangzhou KingMed Diagnostics Group Co., Ltd., (Guangzhou, China) using Simoa assay (Quanterix Co., Billerica, MA, United States), as previously described in detail elsewhere (Rohrer et al., 2016). All measurements were conducted by laboratory technicians who were blinded to the clinical data.

### Statistical Analysis

All data were analyzed using SPSS (version 22.0; IBM, Armonk, NY, United States). The normality of the data was examined using histograms and the Shapiro–Wilk test. Baseline demographic characteristics and other characteristics of the two groups were assessed using the Wilcoxon test, *t*-test, chi-squared test, or Fisher’s test, based on whether the data conformed to a normal distribution or not. The independent sample *t*-test or the Mann–Whitney U test was used to compare the observation indicators between the two groups at the same time point, and the paired sample *t*-test or paired Wilcoxon test was used for pairwise comparisons in the two groups at different time points. Correlation analysis was conducted using Spearman’s correlation. A value of *p* < 0.05 was statistically significant. Most of the data are presented as mean ± standard deviation (SD). The serum NfL levels were not normally distributed. Therefore, non-parametric tests were used for comparison, and the results were recorded as median and interquartile range.

## Results

### Demographics and Clinical Characteristics

Forty-eight participants were screened as eligible for the study and agreed to participate. The participants were randomly divided into two groups: 31 participants were allocated to the active rTMS group, and 17 to the sham rTMS group. Three participants withdrew their consent and quit the study. Thus, 45 participants concluded the rTMS sessions: 30 in the active rTMS group and 15 in the sham rTMS group. The mean age of the registrants was 48.9 ± 10.9 years, and most were male (*n* = 44, 97.8%). All participants were Chinese.

### Baseline Group Differences

No significant differences existed between the treatment and control groups in baseline demographics and clinical characteristics (Table 2). In addition, the mean mental health summary scale of the alcohol-dependent patients was lower than the China population norm (Li et al., 2001).

**TABLE 2:** Demographics and clinical characteristics at baseline.

	Active rTMS group	Sham rTMS group	*p*-value
	(*N* = 30)	(*N* = 15)	
Age	50.50 ± 10.65	45.80 ± 11.14	0.176
Sex, male	29 (96.70)	15 (100.00)	0.475
Years of education	9.97 ± 2.77	10.07 ± 2.84	0.910
Years of drinking	26.03 ± 12.33	20.60 ± 8.32	0.089
*^a^*Heavy drinking days	4.90 ± 1.84	4.47 ± 1.85	0.462
VAS	4.27 ± 2.16	4.33 ± 2.69	0.929
NFL	27.90 (16.94)	30.12 (18.88)	0.682
Vitality	49.33 ± 13.05	48.33 ± 13.84	0.813
Social functioning	43.33 ± 14.21	44.17 ± 12.38	0.848
Role emotional	43.33 ± 26.48	46.66 ± 21.08	0.674
Mental health	40.13 ± 14.27	38.40 ± 13.76	0.699
Mental component summary score	44.03 ± 10.30	44.39 ± 7.70	0.906

*Data are presented as mean ± standard deviation, or median (interquartile range), or n (%). VAS, visual analog scale; NFL, neurofilament light chain. ^a^Heavy drinking days represents Median number of heavy drinking days per week in past 2 weeks.*

### Primary Outcome Analyses

#### Safety and Tolerability of Repetitive Transcranial Magnetic Stimulation

No participant who received rTMS experienced serious adverse medical events. Only two patients reported mild headaches, which worsened after treatment and resolved spontaneously after a few hours. No participant dropped out because of severe adverse effects or an inability to tolerate treatment.

#### Days of Heavy Drinking

We compared the average number of days of heavy drinking per week in the previous 2 weeks at baseline with the average number of days of heavy drinking per week at the end of treatment. Both groups showed a decrease from the baseline level, with a more significant decrease in the treatment group (*p* < 0.01) and a slight decrease in the control group (*p* = 0.04). The reduction was significantly greater in the treatment group than in the control group (*p* < 0.01) (Table 3).

**TABLE 3:** Changes in clinical outcomes for participants receiving active repetitive transcranial magnetic stimulation (rTMS) compared with those receiving sham rTMS.

	Active rTMS group		Sham rTMS group		
	(*N* = 30)		(*N* = 15)		
	Baseline	Change	Baseline	Change	*p*-value*^a^*
*^b^*Heavy drinking days	4.90	–2.37	4.47	–0.53	< 0.001*
VAS	4.27	–1.80	4.33	–0.13	0.001*
NFL	27.23	–5.81	26.48	–1.05	< 0.001*
Vitality	49.33	1.33	48.33	0.67	0.513
Social functioning	43.33	12.92	44.17	4.17	0.007*
Role emotional	43.33	3.33	46.66	2.22	0.719
Mental health	40.13	6.80	38.40	0.93	0.001*
Mental component summary score	44.03	6.10	44.39	2.00	0.005*

Data are presented as mean. VAS, visual analog scale; NFL, neurofilament light chain. ^a^P-value represent the comparation of changes in clinical outcomes between active rTMS group and sham rTMS group. ^b^Heavy drinking days represents Median number of heavy drinking days per week in past 2 weeks. *p < 0.05.

#### Alcohol Craving

The VAS scores were significantly lower after treatment than at the baseline in the treatment group (*p* < 0.01), but no significant changes occurred in the control group (*p* > 0.05). At the end of treatment, the VAS score was significantly lower in the treatment group than in the control group (*p* < 0.01) (Table 3).

#### Serum Neurofilament Light Chain

After the intervention, the serum NfL levels in the treatment group were significantly lower than the levels at baseline (*p* < 0.01), and the serum NfL levels in the control group were also slightly lower than those at baseline (*p* = 0.02). The decrease in serum NfL levels was significantly greater in the treatment group than in the control group (*p* < 0.01) (Table 3).

#### Improvements in the Mental Health Component Score

**FIGURE 1:**
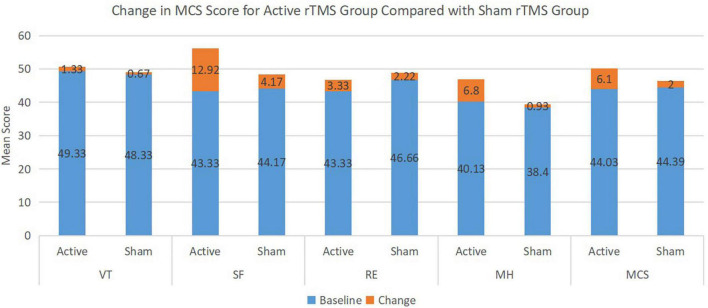
Change in mental health component score (MCS) for active repetitive transcranial magnetic stimulation (rTMS) group compared with sham rTMS group.

Regarding the overall summary scales, the active rTMS group improved significantly more than the sham rTMS group in the mental component summary scales from baseline to the end of the study period (*p* = 0.005) (Table 3). In the individual scales, active rTMS resulted in a significantly greater improvement from baseline in the social functioning scale (*p* = 0.007) and the mental health scale (*p* = 0.0013) than the sham rTMS group. No significant improvement occurred in the vitality or role-emotional scales in either group; thus, no significant differences existed between the scores at the end of the study and baseline (Figure 1).

#### Correlation Between Drinking Patterns and Other Clinical Outcomes

To determine whether changes in drinking patterns could affect other clinical outcomes, we analyzed the correlation between the change in the percentage of heavy drinking days and changes in NfL levels, vitality, social functioning, role-emotional, mental health, and MCS scores. The reduction from the baseline drinking level was positively correlated with the decline in the VAS and NfL levels. However, these reductions were not correlated with vitality or social functioning, role-emotional, mental health, and MCS scores (Table 4).

**TABLE 4:** Change in drinking pattern in relation to changes in clinical outcomes following treatment.

	Percentage days of heavy drinking	
	*r*	*p*-value
VAS	0.553	< 0.001*
NFL	0.837	< 0.001*
Vitality	–0.048	0.754
Social functioning	0.136	0.374
Role emotional	0.106	0.490
Mental health	0.184	0.226
Mental component summary score	0.204	0.179

Statistics in the table represent the correlation between the difference values of each indicator before and after treatment. VAS, visual analog scale; NFL, neurofilament light chain. *p < 0.05.

## Discussion

This study, we assessed the effects of rTMS of the left DLPFC in participants with AUD on serum NfL, alcohol consumption, craving, and psychological impairment. We found that, compared to sham rTMS, active rTMS significantly reduced the number of days of heavy drinking, alcohol craving, serum NfL levels, and improved social functioning and mental health. In addition, the reduction from the baseline drinking level was positively correlated with the declines in VAS score and NfL levels, but not with psychological scores.

Alcohol use disorder is a complex phenomenon with an array of causes and consequences that may affect several aspects of personal life, such as work, relationships, and mental health, and it increases the risk of other problems such as brain damage (Dupuy and Chanraud, 2016). AUD is, unfortunately, a very common disorder in China and around the world. Therefore, the prevention and treatment of AUD is an important public health problem that needs to be solved urgently. These symptoms may be closely associated with dysfunction of the frontocerebellar circuit caused by excessive alcohol consumption (Rogers et al., 2012; Terraneo et al., 2016). TMS is a non-invasive method that allows the magnetic field generated by the coil to effectively pass through the electrically insulated skull, thereby allowing magnetic stimulation to induce an intracranial current in the underlying brain tissue. Reports have indicated that high-frequency rTMS therapy of the left DLPFC can reduce cocaine use and craving levels in patients with cocaine use disorder (Terraneo et al., 2016). The effect of TMS on DLPFC has also been demonstrated in heroin (Liu et al., 2020), methamphetamine (Su et al., 2017) and nicotine (Pripfl et al., 2014) users. Some scholars believe that this phenomenon may be related to the fact that high-frequency rTMS of the left DLPFC can promote neural remodeling of the addiction circuitry (Diana et al., 2017). Interestingly, some researchers have applied this technique to pathological gamblers, considered patients with mental disorders and altered emotional processes (Maniaci et al., 2017) to observe its effect on cue-induced craving (Sauvaget et al., 2018). In addition to the application of TMS in addiction diseases, it also plays an important role in the application of more diseases. The use of TMS in pregnant women with depression is particularly important given the limitations that gestation may have on the tolerability and clinical effectiveness of medications (Hızlı Sayar et al., 2014). It is expected to be more widely used in pregnant women with conditions such as multiple sclerosis (Cuello et al., 2019) and other diseases.

Globally, a high percentage of women consume alcohol during pregnancy (Mårdby et al., 2017), jeopardizing the development of the offspring (Anderson et al., 2014). The teratogenic effect of fetal alcohol exposure is well known. It may lead to potential problems, instantly after birth, at infancy, or even later, and mental impairment in life (Hepper et al., 2005). Similarly, in animal models, the harmful consequences induced by alcohol consumption during gestation and lactation are not limited to the direct *in utero* effects of the drug on the fetus but also extend to maternal care, which contributes to the psycho-behavioral development of the offspring. In particular, the inheritance of alcohol-related mood disorders and vulnerability to alcohol abuse are among the main consequences (Brancato et al., 2016; Brancato and Cannizzaro, 2018). Therefore, there may be a considerable advantage in using rTMS to treat alcohol or other substance addiction during critical periods of brain development. The use of drugs during pregnancy is still a subject of great debate due to the risk of adverse effects. Thus, rTMS, with its unique characteristics of being non-invasive, simple, and safe, may be an acceptable choice for pregnant women who use alcohol.

Serum NfL levels increase with the degree of axonal damage in various neurological conditions such as inflammation, neurodegenerative, traumatic, and cerebrovascular conditions (Gaetani et al., 2019). Some scholars have considered NfL as a disease activity indicator for multiple sclerosis, Parkinson’s disease, and other disorders; however, few reports exist on the application of this index for the efficacy monitoring of alcohol-related brain injury, which is an innovation of this study. In addition, our previous research confirmed that the serum NfL levels of individuals with AUD are higher than those of healthy people. In the current study, we observed changes in the NfL levels in the intervention and control groups before and after treatment with rTMS.

The study demonstrated that 10 daily rTMS sessions over the left DLPFC for 2 weeks in individuals with AUD induced a reduction in the number of days of heavy drinking and alcohol cravings, whereas the control group had a mild reduction in days of heavy drinking. This difference may be related to the fact that participants were asked to stop drinking at the beginning of the treatment. The decrease in heavy drinking days and the VAS score was significantly greater in the active rTMS group than in the sham rTMS group. This finding indicated that rTMS of the left DLPFC could reduce alcohol consumption and drinking desire.

We found that the decrease in serum NfL levels in individuals with AUD was significantly greater in the intervention group than in the control group, which may be the result of a comprehensive effect: excessive alcohol consumption can cause the loss of small white matter fibers, myelin irregularity, and segmental demyelination or remyelination with neuroinflammation (Alfonso-Loeches et al., 2010). In humans and rats, diffusion tensor imaging has revealed that changes occur in early abstinence, which indicates that self-repair of white matter occurs in the early stage of abstinence (De Santis et al., 2019). Based on the positive correlation between the change in drinking patterns and NfL levels, the decline in NfL levels may be due to reductions of alcohol consumption in individuals with AUD; thus, damaged brain tissue can repair itself (West et al., 2019). The regulatory effect of rTMS of the left DLPFC on the addiction circuit accelerates this process.

Repetitive transcranial magnetic stimulation treatment has also been associated with clinically significant improvements in social functioning and mental health. This finding suggests that additional benefits may occur along with abstinence. Improvements in scores also occurred among participants with higher scores at baseline. This study observed that social functioning, mental health, and MCS scores in the intervention group improved significantly more than those in the control group before and after treatment. Moreover, no significant correlation existed between the improvement in scores and the reduction in alcohol consumption. This finding suggested that the effect of rTMS on social functioning and mental health was independent of the reduction in alcohol consumption. These changes in scores may be attributed to the therapeutic effect of rTMS on depression and anxiety.

### Limitations

Aging may be a confounding factor in our longitudinal study because NfL levels also change with normal aging. However, the influence of aging was limited in this study because, instead of comparing NfL levels directly, we compared the changes in NfL levels before and after the intervention. Other limitations included the small sample size and failure to conduct long-term follow-up. The long-term maintenance of abstinence and resuming drinking among the participants requires follow-up. Compared to non-invasive stimulation of other parts of the brain, rTMS of the left DLPFC may have some advantages in improving a negative mood, which may help sustain sobriety, and could be a future research direction.

## Conclusion

In this study, we reported the effect of rTMS of the left DLPFC on the reducing days of heavy drinking, alcohol craving, and serum NfL levels and improving social functioning and mental health in participants with AUD. The results indicated that rTMS of the left DLPFC had good safety and tolerance, reducing alcohol consumption and alcohol craving within a short period in an alcohol-dependent population. In addition, it had a positive impact on neuropsychological and social function. NfL can be used as an early serological indicator of changes in the degree of axonal injury induced by alcohol. In future studies, we will follow the long-term alcohol consumption and mental status of patients with AUD who received left DLPFC intervention. rTMS at the left DLPFC is expected to be an effective treatment method for people who drink excessively with mental health problems.

## Data Availability Statement

The original contributions presented in the study are included in the article/supplementary material, further inquiries can be directed to the corresponding author.

## Ethics Statement

The studies involving human participants were reviewed and approved by the Ethics Committee of First Affiliated Hospital of Zhengzhou University, Zhengzhou, China. The patients/participants provided their written informed consent to participate in this study.

## Author Contributions

YJ designed the experiment. BS participated in the TMS intervention. YL and KW collated the data. RD analyzed the data. ZG recruited the volunteers. TZ wrote the manuscript with substantial contributions from LJ and BM. All authors contributed to the article and approved the submitted version.

## Conflict of Interest

The authors declare that the research was conducted in the absence of any commercial or financial relationships that could be construed as a potential conflict of interest.

## Publisher’s Note

All claims expressed in this article are solely those of the authors and do not necessarily represent those of their affiliated organizations, or those of the publisher, the editors and the reviewers. Any product that may be evaluated in this article, or claim that may be made by its manufacturer, is not guaranteed or endorsed by the publisher.
